# Novel Blood Biomarkers for a Diagnostic Workup of Acute Aortic Dissection

**DOI:** 10.3390/diagnostics11040615

**Published:** 2021-03-30

**Authors:** Anja Forrer, Felix Schoenrath, Michael Torzewski, Jens Schmid, Urlich F. W. Franke, Nora Göbel, Drahomir Aujesky, Christian M. Matter, Thomas F. Lüscher, Francois Mach, David Nanchen, Nicolas Rodondi, Volkmar Falk, Arnold von Eckardstein, Joanna Gawinecka

**Affiliations:** 1 Institute of Clinical Chemistry, University Hospital of Zurich, University of Zurich, 8091 Zurich, Switzerland; anja.forrer@hispeed.ch (A.F.); arnold.voneckardstein@usz.ch (A.v.E.); 2 Department of Cardiothoracic and Vascular Surgery, German Heart Center Berlin, 13353 Berlin, Germany; schoenrath@dhzb.de (F.S.); Falk@dhzb.de (V.F.); 3 DZHK (German Centre for Cardiovascular Research), Partner Site Berlin, 10785 Berlin, Germany; 4 Department of Laboratory Medicine and Hospital Hygiene, Robert Bosch Hospital, 70376 Stuttgart, Germany; Michael.Torzewski@rbk.de (M.T.); jens.schmid@rbk.de (J.S.); 5 Department of Cardiovascular Surgery, Robert Bosch Hospital, 70376 Stuttgart, Germany; ulrich.franke@rbk.de (U.F.W.F.); nora.goebel@rbk.de (N.G.); 6 Department of General Internal Medicine, Inselspital, Bern University Hospital, University of Bern, 3010 Bern, Switzerland; drahomir.aujesky@insel.ch (D.A.); Nicolas.Rodondi@insel.ch (N.R.); 7 Department of Cardiology, University Heart Center, University Hospital Zurich, University of Zurich, 8091 Zurich, Switzerland; christian.matter@usz.ch (C.M.M.); thomas.luescher@zhh.ch (T.F.L.); 8 Department of Cardiology, University Hospital Geneva, 1205 Geneva, Switzerland; Francois.Mach@hcuge.ch; 9 Center for Primary Care and Public Health, University of Lausanne, 1015 Lausanne, Switzerland; david.nanchen@unisante.ch; 10 Institute of Primary Health Care (BIHAM), University of Bern, 3012 Bern, Switzerland; 11 Department of Cardiothoracic Surgery, Charité–Universitätsmedizin Berlin, corporate member of Freie Universität Berlin, Humboldt-Universität zu Berlin, Berlin Institute of Health, 10117 Berlin, Germany; 12 Department of Health Sciences and Technology, ETH Zurich, 8092 Zurich, Switzerland

**Keywords:** acute aortic dissection, biomarker, D-dimers, pulmonary embolism, acute myocardial infarction, IL-10

## Abstract

Acute aortic dissection (AAD) is a rare condition, but together with acute myocardial infarction (AMI) and pulmonary embolism (PE) it belongs to the most relevant and life-threatening causes of acute chest pain. Until now, there has been no specific blood test in the diagnostic workup of AAD. To identify clinically relevant biomarkers for AAD, we applied Proseek^®^ Multiplex assays to plasma samples from patients with AAD, AMI, PE, thoracic aortic aneurysm (TAA), and non-cardiovascular chest pain (nonCVD). Subsequently, we validated top hits using conventional immunoassays and examined their expression in the aortic tissue. Interleukin 10 (IL-10) alone showed the best performance with a sensitivity of 55% and a specificity of 98% for AAD diagnosis. The combination of D-dimers, high-sensitive troponin T (hs-TnT), interleukin 6 (IL-6), and plasminogen activator inhibitor 1 (PAI1) correctly classified 75% of AAD cases, delivering a sensitivity of 83% and specificity of 95% for its diagnosis. Moreover, this model provided the correct classification of 77% of all analyzed cases. Our data suggest that IL-10 shows potential to be a rule-in biomarker for AAD. Moreover, the addition of PAI1 and IL-6 to hs-TnT and D-dimers may improve the discrimination of suspected AAD, AMI, and PE in patients presenting with acute chest pain.

## 1. Introduction

Acute chest pain is one of the most frequent reasons for admission to emergency departments [1,2]. A broad variety of thoracic, visceral, as well as psychiatric disorders may cause chest discomfort or pain. As some of these disorders are associated with a high mortality, prompt diagnosis is of the utmost importance for further triage and the timely treatment of patients. In this regard, particular attention should be given to acute myocardial infarction (AMI), pulmonary embolism (PE), and acute aortic dissection (AAD), as they all need rapid intervention [3]. Among these, AAD has the highest mortality rate and its treatment differs substantially from the other two diagnoses. Alongside a patient’s medical history, physical examination, electrocardiogram (ECG), and imaging modalities, laboratory testing of cardiac troponins and D-dimers plays an important role in the diagnosis of “big three” causes of chest pain.

AAD is a rare, but often fatal condition. It emerges from an initial tear in the intima layer of the aortic wall, followed by a splitting of the medial layer and the formation of a false lumen caused by high arterial pressure and pulsatile blood flow within the aortic wall. Risk factors for AAD include pre-existing aortic aneurysm, arterial hypertension, male sex, higher age, and some inherited connective tissue diseases [4,5]. The diagnosis of AAD is often challenging. Retrospective analyses of patients with confirmed aortic dissection suggest that emergency physicians suspect AAD in only half of cases [6,7].

To date, no specific and easily accessible laboratory test for AAD is available. Some guidelines suggest D-dimers for the ruling out of AAD [8]. However, D-dimers lack specificity as they are also elevated in patients with PE, AMI, and many other acute diseases [9]. We therefore aimed to identify novel biomarker candidates that could improve the diagnostics of AAD among patients who present with acute chest pain. For that reason, we applied a Proseek^®^ Multiplex assay of 354 predefined proteins to plasma samples from patients with either AAD, AMI, PE, thoracic aortic aneurysm (TAA), or non-cardiovascular chest pain (nonCVD). Five top hits were then validated using the conventional immunoassays and their diagnostic performance for AAD was compared with high sensitivity cardiac troponin T (hs-TnT) and D-dimers.

## 2. Material and Methods

### 2.1. Patients

This study was performed at the University Hospital Zurich in accordance with the Declaration of Helsinki, and with the approval of the Cantonal Ethics Committee in Zurich, Switzerland (KEK-ZH-Nr. 2013–0047 and KEK-ZH-Nr. 2016–00378), Ethics Committee of the University of Tübingen, Germany (465/2017BO2), and Ethics Committee of the Charité–University Medical Centre Berlin, Germany (EA 2/2018/18).

Consecutive patients diagnosed with acute aortic dissection type A (AAD), thoracic aortic aneurysm (TAA), and aortic valve replacement (AVR) were recruited at the Department of Cardiovascular Surgery of the University Hospital Zurich. Inclusion criteria comprised both sexes, age ≥ 18 years, the presentation of acute aortic syndrome, or hospital admission for elective surgery of TAA or AVR. Median estimated time interval from the onset of symptoms to diagnosis was 5 h for AAD patients, and all AAD patients were diagnosed within the first 24 h from the onset of symptoms. All AAD patients were diagnosed by means of computer tomography and underwent aortic graft replacement. Patients who were unable to comprehend the study or provide voluntary informed consent (i.e., due to cognitive impairment), and those who had had previous aortic intervention (e.g., aortic graft placement), were excluded.

The plasma samples from seven patients diagnosed with AAD type A were collected from the DZHK (Deutsches Zentrum für Herz-Kreislaufforschung) biobank of the Charité–University Medical Centre Berlin, Germany. Inclusion criteria comprised both sexes, age ≥18 years, the presentation of with AAD, and written informed consent for the use of biobank samples.

Non-cardiovascular chest pain control patients (nonCVD) were recruited at the Emergency Department of the University Hospital Zurich from patients who presented with acute chest pain, a leading symptom of AMI, PE, or AAD, and received a final diagnosis of non-cardiovascular diseases [10]. In most cases, chest pain was caused by musculoskeletal, dyspeptic, or psychiatric factors such as anxiety or panic attack. Inclusion criteria comprised both sexes, age ≥ 18 years, and chest pain or discomfort as a leading symptom. Patients who were unable to comprehend the study or provide voluntary informed consent (i.e., due to cognitive impairment) were excluded.

Symptomatic patients with acute myocardial infarction (AMI) were selected from the SPUM-ACS cohort (ClinicalTrials.gov (accessed on 19 February 2021) Identifier: NCT01000701) [11]. General inclusion criteria comprised both sexes, age ≥ 8 years, presenting within five days (preferably within 72 h) after onset of pain with a main diagnosis of STEMI, NSTEMI, or unstable angina. Exclusion criteria comprised severe physical disability, inability to comprehend the study, or less than one year of life expectancy for non-cardiac reasons.

Patients with symptomatic pulmonary embolism (PE) were selected from The Swiss Venous Thromboembolism Cohort 65+ (SWITCO-65+) conducted in nine Swiss study centers (ClinicalTrials.gov (accessed on 19 February 2021); NCT00973596). Symptomatic PE was defined as a positive spiral computed tomography or in pulmonary angiography, high-probability ventilation-perfusion scan, or proximal deep vein thrombosis documented by compression ultrasonography or contrast venography in patients with acute chest pain, new or worsening dyspnea, haemoptysis, or syncope. Exclusion criteria included the inability to provide informed consent (i.e., due to severe dementia), conditions incompatible with follow-up (i.e., terminal illness or location too far away from the study center), insufficient German or French-speaking ability, thrombosis at sites other than the lower limbs, and catheter-related thrombosis [12].

In general, blood samples where obtained from consenting patients before any intervention at the time of diagnosis, centrifuged in the laboratory, and stored as plasma at −80 °C until further analysis.

If surgically feasible, aortic tissue samples from consenting patients were collected during open-chest surgery for AAD type A, TAA, or AVR. After placing in a sterile ice-cold PBS buffer, resected aortic tissue samples were transferred to the laboratory as soon as possible, snap-frozen, and stored at −80 °C or embedded in paraffin until further analysis.

Patients for the following study were selected from the above-mentioned cohorts based on sex and age, arbitrarily divided in two cohorts: (1) exploratory cohort for the analysis by Proseek^®^ Multiplex assay, and (2) confirmatory cohort for the validation of selected biomarkers with conventional immunoassays. Due to the limited availability of plasma samples, we included samples of 81 random patients from the exploratory cohort into the confirmatory cohort (*n* = 184): 23 out of 34 AAD (68%), 18 out of 35 TAA (51%), 30 out of 46 AMI (65%), 7 out of 36 PE (19.5%), and 3 out of 33 nonCVD (9%).

The detailed descriptions of the cohorts are provided in Table 1 and Table 2.

### 2.2. Multiplex Biomarker Analysis with PROSEEK^®^ Multiplex Assay

One-hundred-and-fifty heparin plasma samples of patients from the exploratory cohort were analyzed using the OLINK Proseek^®^ Multiplex Cardiovascular II and III (CVD II and CVD III), Inflammation (INF I), and Oncology II (ONC II) panels (Olink Proteomics, Uppsala, Sweden). OLINK Proseek^®^ Multiplex technology is based on proximity extension assay that uses matching pairs of antibodies linked to unique oligonucleotides for the detection and real-time PCR quantification of predefined 92 biomarker candidates per panel [13]. For the statistical analysis, we only included proteins with concentrations above the level of detection in more than 75% of the plasma samples, namely, 88 (96%), 92 (100%), 68 (74%), and 92 (100%) proteins assayed with the CVD II, CVD III, INF I, and ONC II panels, respectively.

The obtained data are presented as normalized protein expression (NPX) values (i.e., arbitrary units). Values below the limit of detection were replaced with the value for the limit of detection for further statistical analysis.

### 2.3. Single Biomarker Analyses

The hs-TnT (Elecsys Troponin T hs), IL-6 (Elecsys IL-6), D-dimers (Tina-quant D-Dimer), and CPR were centrally measured in the Institute of Clinical Chemistry of the University Hospital Zurich by fully automated immunoassays developed for the Cobas 8000 e602 or the c501 modular analyzer (Roche Diagnostics, Rotkreuz, Switzerland). IL-10 was measured with a bead-based Human IL-10 Flex Set immunoassay (BD Biosciences, San Jose, CA, USA) on a Navios Flow Cytometer (Beckman Coulter, Indianapolis, IN, USA). Insulin-like growth factor binding protein 1 (IGFBP1), plasminogen activator inhibitor 1 (PAI1), and interleukin 1 receptor antagonist (IL-1ra) were measured on a DS2 ELISA Processor (Dynex Technologies, Chantilly, VA, USA) using a Human IGFBP-1 ELISA Kit (Sigma-Aldrich, Buchs, Switzerland), Human Total Serpin E1/PAI1 QKit, and IL-1ra/IL-1F3 Quantikine Kit (both from R&D Systems, Minneapolis, MN, USA), respectively. All reagents, tests, and control materials were stored and used according to the manufacturer’s instructions.

### 2.4. RNA Sequencing

RNA was isolated from the aortic tissue samples of eight patients with Stanford type A AAD, seven with TAA, and six with aortic valve stenosis (AVR) by using the NucleoSpin miRNA kit for miRNA and RNA purification (Macherey-Nagel, Düren, Germany). The quality of the isolated RNA was determined with a NanoDrop 2000 UV–Vis spectrophotometer (ThermoFisher Scientific, Waltham, MA, USA) and a Bioanalyzer 2100 (Agilent, Waldbronn, Germany). Only samples with a 260 nm/280 nm ratio between 1.8–2.1 and a 28S/18S ratio within 1.5–2 were further processed. The TruSeq RNA Sample Prep Kit v2 (Illumina Inc., San Diego, CA, USA) was used for the subsequent steps. Briefly, 300 ng of total RNA samples were ribo-depleted using Ribo Zero Gold (Epicentre^®^, San Diego, CA, USA) and then fragmented. The fragmented samples were reverse transcribed to cDNA, end-repaired, and polyadenylated before ligation of TruSeq adapters containing the index for multiplexing. Fragments containing TruSeq adapters on both ends were selectively amplified with PCR. The quality and quantity of the enriched libraries were validated using a Qubit^®^ 1.0 Fluorometer (Life Technologies, Carslbad, CA, USA) and a Caliper GX LabChip^®^ GX (Caliper Life Science Inc, Waltham, MA, USA). The product was a smear with an average fragment size of approximately 260 bp. The libraries were normalized to 10 nM in Tris-HCl 10 mM, pH 8.5 with 0.1% Tween 20. The TruSeq PE Cluster Kit v4-cBot-HS or TruSeq SR Cluster Kit v4-cBot-HS (Illumina Inc., California, CA, USA) was used for cluster generation using 10 pM of pooled normalized libraries on the cBOT. Sequencing were performed on the Illumina HiSeq 2500 paired end at 2 X101 bp or single end 100 bp using the TruSeq SBS Kit v4-HS (Illumina Inc., San Diego, CA, USA).

### 2.5. Histological and Immunohistochemical Staining

Serial 3 µm thick sections of paraffin-embedded aortic samples from 15 Stanford type A AAD, four TAA, and four AVR patients were deparaffinized and stained with hematoxylin and eosin (H&E) or Elastica van Gieson (EvG) stain. Immunohistochemistry was performed using the Dako REAL EnVision Detection System, rabbit/mouse kit (K5007, DakoCytomation, Glostrup, Denmark), and treated with 0.3% Peroxidase Block (DakoCytomation, Glostrup, Denmark) to block endogenous peroxidase activity, and then blocked with the host serum of a secondary antibody. After blocking, slides were incubated with the following primary antibodies for 60 min: mouse monoclonal IL-1ra (Santa Cruz Biotechnology, Dallas, TX, USA), mouse monoclonal Serpine1/PAI1 (Covalab, Villeurbanne, France), mouse monoclonal human platelet glycoprotein IIIa/CD61 (Diagnostic BioSystems, Pleasanton, CA, USA), mouse monoclonal CD68 (DakoCytomation, Glostrup, Denmark), CD163 (Cell Marque Corporation, Rocklin, CA, USA), rabbit polyclonal anti-interleukin-6 antibody (antibodies-online GmbH, Aachen, Germany), rabbit monoclonal antibody CD3 (Cell Marque Corporation, Rocklin, CA, USA), mouse monoclonal antibody CD19 (Cell Marque Corporation, Rocklin, CA, USA), rabbit polyclonal anti-IL-10 antibody (Abcam, Cambridge, UK), and rabbit polyclonal anti-insulin-like growth factor binding protein-1 (antibodies-online GmbH, Aachen, Germany). Antigen-retrieval of the primary antibodies was achieved by heating the sections in a target retrieval solution (pH 9; DakoCytomation, Glostrup, Denmark) in a steamer for 30 min. The application of the primary antibody was followed by incubation with the secondary antibody for 30 min. The reaction products were revealed by immersing the slides in diaminobenzidine tetrachloride (DAB) to give a brown reaction product. Finally, slides were counterstained with hematoxylin (Merck Millipore, Burlington, MA, USA) and mounted. The positive staining of the following tissues was confirmed in order to demonstrate the suitability of the antibodies used: breast cancer for Il-6, kidney for IGFB1, esophagus for IL-1ra, colon for IL-10, and pancreas for PAI1.

The characteristics of the antibodies and images of the positive staining are provided in Supplementary Table S1.

### 2.6. Statistical Analysis

Statistical analyses were performed using SPSS 26.0 for Windows (IBM Software, New York, NY, USA). Results are reported either as means ± SD for normally distributed data or as medians with 10th and 90th percentiles in brackets for the not normally distributed data. Continuous variables that were not normally distributed, as determined using the Kolmogorov–Smirnov test, were logarithmically transformed before analysis in order to resemble a normal distribution. Categorical variables were compared using the χ^2^ test. One-way ANOVA with Hochberg correction was used for multiple comparisons of OLINK Proseek^®^ biomarkers between analyzed groups in the exploratory cohort. One-way ANOVA with Games–Howell correction was used for multiple comparisons of biomarkers between analyzed groups in the confirmatory cohort. ROC curve analyses were applied to explore the diagnostic performance of biomarkers to express the area under the curve (AUC), sensitivity, and specificity. Discriminant function analysis was applied for biomarker combinations to explore their power to differentiate AAD from other diagnoses and for the overall classification of patients by the correct diagnosis. Only patients with all parameters employed in certain models were included in the discriminant analysis. All reported *p*-values are two-tailed and *p* < 0.05 indicates statistical significance.

## 3. Results

### 3.1. Exploratory Multiplex Biomarker Analyses

In total, 354 unique proteins were analyzed in 150 plasma samples of patients from the exploratory cohort using the Proseek^®^ Multiplex Cardiovascular II and III (CVD II and CVD III), Inflammation (INF I), and Oncology II (ONC II) panels. AAD patients differed significantly from at least one other disease (nonCVD, TAA, AMI, or PE) by plasma concentrations of different 181 proteins (Supplementary Table S2). Among them, plasma concentrations of seven proteins showed significant differences between AAD and all other diagnoses: interleukin-1 receptor antagonist protein (IL-1ra), interleukin 10 (IL-10), interleukin-1 receptor-like 2 (IL-1RL2), interleukin 6 (IL-6), insulin-like growth factor-binding protein 1 (IGFBP1), plasminogen activator inhibitor 1 (PAI1), and lymphotoxin-alpha (TNFB). In AAD, the median protein concentration of IL-6 was 12–15 times higher when compared to nonCVD and TAA, 6–7 times higher when compared to AMI, and twice as high when compared to PE. Compared to all other diagnoses, the median protein concentrations in AAD plasmas were six to seven times higher for IL-10, two to three times higher for IL-1ra and PAI1, and only one and half times higher for IL-1RL2, but up to two times lower for TNFB (Table 3 and Supplementary Figure S1).

### 3.2. Confirmatory Single Biomarker Analyses

**Figure 1:**
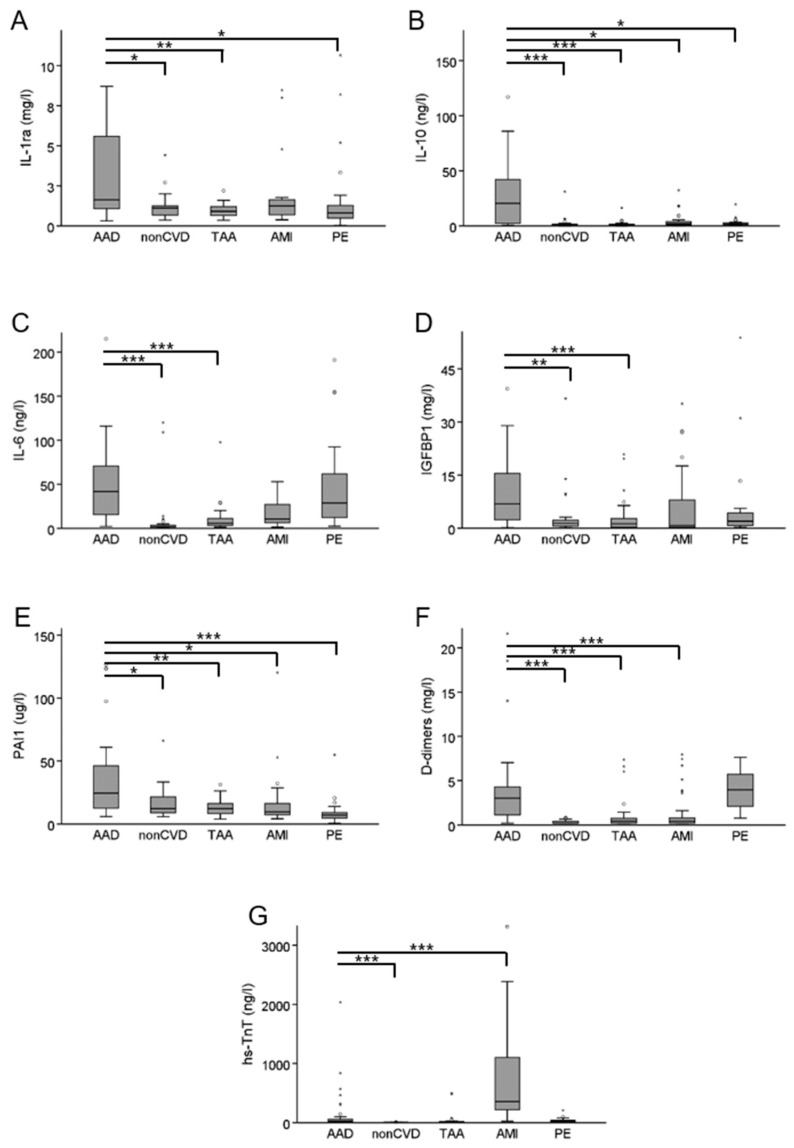
Plasma concentrations of IL-1ra (panel **A**), IL-10 (panel **B**), IL-6 (panel **C**), IGFBP1 (panel **D**), PAI1 (panel **E**), D-dimers (panel **F**), and hs-TnT (panel **G**) in the confirmatory cohort. * *p*-value < 0.05; ** *p*-value < 0.01, *** *p*-value < 0.001.

We next aimed for the confirmation of results obtained with the multiplex assays for IL-1ra, IL-10, IL-6, IGFBP1, and PAI1 by using conventional immunoassays in the confirmatory cohort (Table 2). In general, the results of the exploratory analyses were confirmed to a large extent. The IL-1ra median concentration of 1.6 mg/L in AAD was marginally but significantly higher than in nonCVD (1.1 mg/L), TAA (0.9 mg/L), or PE (0.8 mg/L), while no significant difference was found in comparison with AMI (1.2 mg/L). Very striking differences were found for IL-10, which had median concentrations of 20.6 ng/L in AAD but only of 0.6 ng/L in nonCVD, 0.7 ng/L in PE, 0.9 ng/L in TAA, and 1.9 ng/L in AMI. Further, the median concentration of PAI1 was higher in AAD (24.3 µg/L) than in all other groups (nonCVD 12.2 µg/L, TAA 12.2 µg/L, AMI 9.6 µg/L, and PE 7.0 µg/L). IL-6 was present in higher median concentrations in AAD (41.8 ng/L) when compared to nonCVD (1.8 ng/L) and TAA (5.8 ng/L), but not in comparison to AMI (10.6 ng/L) and PE (28.8 ng/L). The IGFBP1 median concentration in AAD (6.9 mg/L) was higher than in nonCVD (1.4 mg/L), TAA (1.2 mg/L), and AMI (0.8 mg/L), but not when compared to PE (2.0 mg/L). Detailed results are provided in Figure 1 and Supplementary Table S3.

ROC curve analyses were applied to estimate the diagnostic performance of biomarkers. The area under the curve (AUC) for AAD diagnosis reached only 0.51 and 0.76 for hs-TnT and D-dimers, respectively. At the threshold of 0.3 mg/L, D-dimers reached a sensitivity of 97% and specificity of 38% for AAD diagnosis. While at a generally accepted threshold for AAD diagnosis of 0.5 mg/L, sensitivity reached 94% and specificity 42%. Overall, the highest AUC of 0.83 was found for IL-10, with a sensitivity and specificity of 55% and 98%, respectively, at the threshold of 20 ng/L. In comparison, D-dimers could reach a sensitivity of 55% at the cost of lower specificity of 75% at the threshold of 2 mg/L, and specificity of 98% at the cost of very low sensitivity of 9% at the threshold of 7.5 mg/L. With an AUC of 0.78, PAI1 showed the second-best diagnostic performance with a sensitivity and specificity of 48% and 97%, respectively, at threshold of 58 µg/L. The other biomarkers showed similar or worse performances than the D-dimers. The details of the ROC curve analysis are provided in Table 4 and Supplementary Figure S2.

The discriminant function analysis was applied to all possible combinations of two, three, or four biomarkers to explore their power to separate AAD from other patient groups. The combination of the two established biomarkers, namely hs-TnT and D-dimers, grouped correctly 26% (9 out of 34) of AAD cases and 60% (109 out of 181) of all diagnoses (AAD, PE, AMI, TAA, and nonCVD). The combination of D-dimers, IL-6, and PAI1 correctly classified 75% (15 out of 20) of AAD cases and 60% (73 out of 122) of all diagnoses. The introduction of hs-TnT into the latter model improved overall classification to 77% (94 out of 122) (Table 5). All other models showed worse discrimination power (data not shown).

### 3.3. Expression of Biomarker Candidates in Aortic Tissues

Finally, we used mRNA sequencing and immunohistochemistry to investigate the mRNA and protein expression, respectively, of our biomarker candidates in aortic tissue. Since the sampling of aortic tissue from living patients is only possible during open-chest cardiovascular surgery, patients undergoing aortic valve replacement (AVR) were chosen as the best possible control tissue. As a second control group, we investigated aortic tissues from TAA patients.

The mRNA expression of IL-1ra, IL-10, IL-6, and PAI1 was higher in AAD than in both TAA and AVR, while no difference in mRNA expression was found for IGFBP1 (Supplementary Table S4).

**Figure 2:**
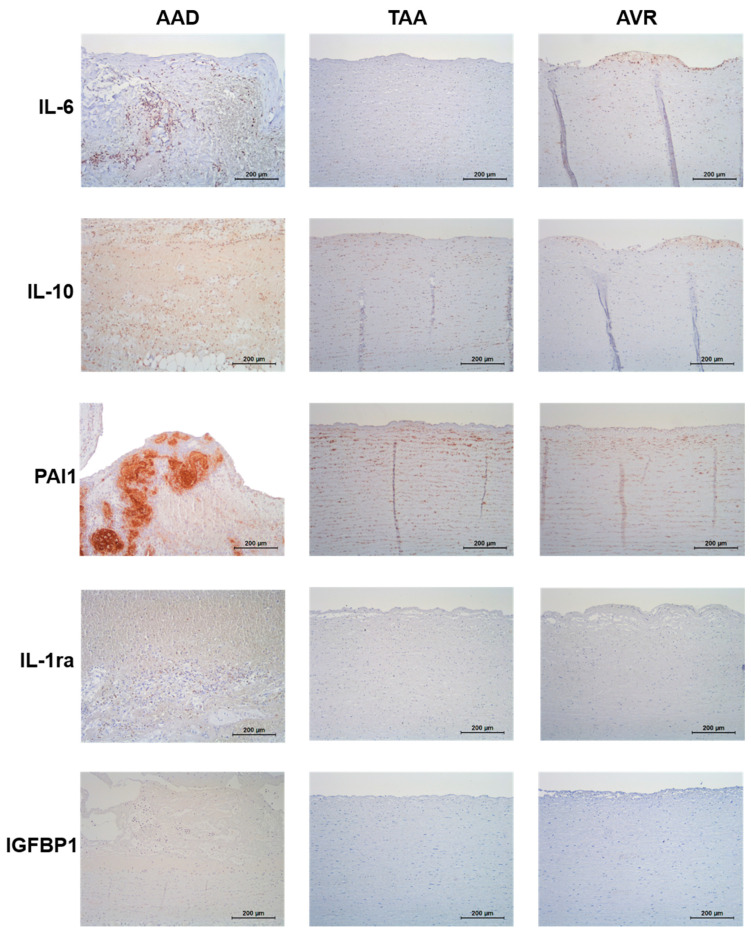
Immunohistochemical staining of IL-6, IL-10, PAI1, IL-1ra, and IGFBP1 in the aortic tissue of patients with AAD, TAA, and AVR.

The aorta of AAD patients contained IL-10-positive cells surrounded by a plethora of erythrocytes. Additional immunostaining demonstrated that these cells were granulocytes and both CD3+ and CD19+ lymphocytes. The control tissue specimen from patients with either TAA or AVR showed faint IL-10 staining of smooth muscle cells or initial atherosclerotic lesions. In AAD, the IL-6 staining was very similar to that of IL-10. Both control tissue specimens were either free of anti-IL-6-immunoreactivity or showed faint IL-6 staining of initial atherosclerotic lesions. Aortas of AAD patients contained IL-1ra-positive cells surrounded by a plethora of erythrocytes. Similar to the IL-6 staining, both control tissue specimen were either free of anti-IL-1ra-immunoreactivity or showed faint IL-1ra staining of initial atherosclerotic lesions. The immunohistochemistry of PAI1 showed festoon-like intense staining within ruptured areas of the aortic wall. Complementary staining demonstrated that these areas were platelet- and fibrin-rich. In both control tissue specimens only faint PAI1 staining of smooth muscle cells was visible. IGFBP1 was detected in all three analyzed tissue specimens but no difference in anti-IGFBP1-immunoreactivity was detected in the dissected tissue (Figure 2 and Supplementary Figure S3).

## 4. Discussion

Alongside AMI and PE, AAD is one of the “big three” underlying conditions of acute chest pain at the emergency department. AAD is a rare condition which is frequently misdiagnosed. Missed or delayed diagnosis contributes to the persistently high AAD mortality rate [3]. Nuclear magnetic resonance imaging, computed tomography, or transesophageal echocardiography are standard methods to confirm the diagnosis of the aortic dissection [14]. However, there is no established blood-derived biomarker for AAD diagnosis, similar to cardiac troponins for AMI [15] or D-dimers for PE [16]. Although D-dimers were proposed as rule-out testing for suspected AAD, they seem to be useful only in low-risk patients and are not recommended for patients with a high probability of AAD [8,17].

In this pilot study, we employed the Proseek^®^ Multiplex biomarker discovery platform with subsequent confirmation with single protein immunoassays. We also investigated the expression of biomarker candidates in aortic tissue on protein and mRNA levels to strengthen the evidence of their specificity through biological plausibility. Briefly, we can report two main findings: (1) IL-10 alone showed better performance than D-dimers in AAD diagnosis, and (2) PAI1, IL-6, high-sensitive troponin T (hs-TnT), and D-dimers as a biomarker panel have the potential to improve the simultaneous discrimination of suspected AAD, AMI, and PE in patients presenting with acute chest pain at the emergency department.

Out of 354 predefined inflammatory, cardiovascular, or oncological biomarker candidates, six proteins were present in plasmas of AAD patients at significantly higher concentrations than in plasmas of patients with one of the most relevant differential diagnoses (AMI, PE, or non-cardiovascular pain) and with the most relevant risk factor (TAA). Among them, five biomarkers were chosen for the confirmatory analysis: interleukin 1 receptor antagonist protein (IL-1ra), interleukin 10 (IL-10), interleukin 6 (IL-6), insulin-like growth factor-binding protein 1 (IGFBP1), and plasminogen activator inhibitor 1 (PAI1). Apart from IGFBP1, all of them were also detected at higher protein or mRNA expression levels in aortic tissues of AAD patients in comparison with those of TAA or AVR patients. Generally, our biomarker candidates reflect pathological processes, such as inflammation [18], systemic activation of coagulation, and fibrinolysis [19,20], which all occur in AAD, either during the development of aortic dissection or in response to the event.

Elevated levels of IL-6 in the peripheral blood of AAD patients have already been reported [18,21,22,23]. Plasma levels of IL-10 were also previously found to be increased in the peripheral blood of AAD patients [22]. IL-10 is an immuno-regulatory cytokine with profound anti-inflammatory functions to limit the excessive tissue disruption caused by an inflammation. The rare IL-10-1082 polymorphism in the promoter region of the IL-10 gene, which is associated with lower levels of the circulating IL-10, is more common in patients with abdominal aortic aneurysm (AAA) and a genetic risk factor for its development [24,25,26]. In a transgenic mouse model, the augmentation of the systemic IL-10 expression reduced both the development of AAA and the rate of dissecting aneurysm [27]. IL-1ra interferes with the pro-inflammatory cytokines of the IL-1 family, which can stimulate gene expression of clotting factors and inhibitors of fibrinolysis [28]. In IL-1ra knock-out mice, angiotensin II induces both development of AAA and aortic inflammation [29]. Interestingly, IL-10 suppresses gene expression of IL-1 [30], pointing to a possible common reason for the increased expression in aortic tissue as well as the elevated plasma levels of IL-10 and IL-1ra in AAD. PAI1 is a primary inhibitor of tissue-type plasminogen and urokinase-type plasminogen activator. PAI1 suppresses the fibrinolysis of blood clots, but also promotes cell adhesion and spreading. Similar to our study, Schneiderman et al. found increased PAI1 gene expression in smooth muscle cells surrounding thrombi in the aortas of AAD patients compared to non-dissected parts of the aortic wall [31]. IGFBP1 prolongs the half-life of insulin-like growth factors and alters their interactions with their surface receptors. Plasma levels of circulating IGFBP1 correlate with an increased diameter of the abdominal aorta in older men with AAA [32] and have already been proposed as a potential biomarker for this condition [33]. However, no association of IGFBP1 with AAD has been reported up to now.

With an AUC of 0.83, IL-10 showed the best performance for AAD diagnosis of all the biomarkers including D-dimers, which had an AUC of 0.76. These two biomarkers differ markedly by their sensitivity and specificity. At a threshold of 0.3 mg/L, D-dimers reached an excellent sensitivity of 97%, but a limited specificity of 38%. While IL-10 at a threshold of 20 ng/L showed a moderate sensitivity of 55% but a superior specificity of 98%. Set side by side, D-dimers could reach a sensitivity of 55% at the cost of lower specificity of 75% at the threshold of 2.0 mg/L, and a specificity of 98% at the cost of the very low sensitivity of 9% at the threshold of 7.5 mg/L. In agreement with the very good sensitivity reported for AAD diagnosis, it is recommended that D-dimers are used for the ruling out of AAD, at least in patients with a low pretest probability [8]. Given its high specificity observed in our study, IL-10 has the potential to be used as a rule-in biomarker. The diagnostic performance of biomarkers can be usually improved by the combination of several markers. In our study, for instance, the addition of IL-6 and PAI1 to hs-TnT and D-dimers in the discriminant function analysis significantly improved both the correct separation of AAD cases from all diagnoses (26% vs. 75%) as well as the correct classification of all diagnoses (60% vs. 77%). The last model reached a satisfying sensitivity of 83% and a specificity of 95% for AAD diagnosis.

One strength of our rather small pilot study is the inclusion of several control groups reflecting the most important differential diagnoses of AAD, (AMI, PE, and nonCVD) as well as patients with a high risk of developing AAD (TAA). Another strength is the replication of our discoveries by independent analytical methods, however in a cohort, which overlaps by 44% with the exploratory cohort. Another drawback potentially influencing our results is the fact that on average PE patients were older and the nonCVD patients younger than rest of patients with AAD, AMI, or TAA. The small sample size per group can be seen as a major limitation. A large-scale study, ideally including AAD at its normal rare incidence rate relative to the much more frequent alternative diagnoses (AMI, PE, or nonCVD), and in several centers of different geographic areas, is needed to confirm our results.

In general, in order for any biomarker to be clinically useful in emergency settings it has to be measured using a rapid and precise assay. For emerging biomarkers in the initial validation phase, such assays are usually not available and are only developed once a biomarker proves its clinical utility in retrospective studies.

In conclusion, IL-10 displays potential to be a rule-in biomarker for AAD diagnosis, and the addition of PAI1 and IL-6 to hs-TnT T and D-dimers has the potential to improve the simultaneous diagnostics of suspected AAD, AMI, and PE in patients with acute chest pain.

## Figures and Tables

**Table 1:** Patients’ characteristics in the exploratory cohort.

Characteristic	Acute Aortic Dissection (AAD) *n* = 29	Non-Cardiovascular Chest Pain Control Patients (nonCVD) *n* = 30	Thoracic Aortic Aneurysm (TAA) *n* = 30	Acute Myocardial Infarction (AMI) *n* = 31	Pulmonary Embolism (PE) *n* = 30	*p*-Value
Age (years)	64 ± 13	63 ± 12	63 ± 13	65 ± 10	78 ± 8	0.068
Sex (males)	21 72%	19 63%	24 80%	21 66%	14 44%	0.134
Hypertension	20 69%	14 47%	17 57%	17 55%	22 73%	0.357
Current smoking	8 28%	5 17%	12 40%	12 39%	1 3%	0.06
Diabetes mellitus	1 3%	4 13%	0 0%	0 0%	3 10%	0.057
Thoracic aortic aneurysm	16 55%	n.d.	30 100%	n.d.	n.d.	n.d.
Bicuspid aortic valve	5 17%	n.d.	5 25% ^§^	n.d.	n.d.	n.d.
hs-TnT [ng/L] ^¥^	18 (5.0–1122)	6.5 (<5.0–16) ^+^	7.9 (<5.0–38) ^++^	279 (104–1768)	16 (5–161)	<0.001
D-dimers [mg/L] ^¥^	3.5 (0.5–13.8)	0.2 (<0.2–0.7) ^$^	0.3 (<0.2–4.4) ^$$^	0.3 (<0.2–3.9) ^$$$^	4.7 (1.4–14.6)	<0.001
CRP [mg/L]	5.0 (0.6–92.3)	2.0 (0.4–26.4)	1.0 (0.3–25.0)	3.6 (0.5–41.1)	58.0 (1.9–172.1)	<0.001

For categorical variables, the *p*-value was determined using the χ^2^ test; for continuous variables, using one-way ANOVA. ^§^ data not available for 33% of patients; ^+, ++^ hs-TnT not detectable (<5 ng/L) in 45% of nonCVD and 31% of TAA patients, respectively; ^$, $$, $$$^ D-dimers not detectable (<0.2 mg/L) in 33% of nonCVD, 19% of TAA, and 22% of AMI patients, respectively; ^¥^ median (10–90th percentile); n.d. not determined.

**Table 2:** Patients’ characteristics in the confirmatory cohort.

Characteristic	AAD *n* = 34	nonCVD *n* = 33	TAA *n* = 35	AMI *n* = 46	PE *n* = 36	*p*-Value
Age (years)	63 ± 13	53 ± 10	63 ± 13	68 ± 11	72 ± 6	<0.001
Sex (males)	27 79%	19 58%	28 80%	27 59%	28 78%	0.052
Hypertension	23 68%	10 30%	18 51%	29 63%	25 69%	0.019
Current smoking	8 24%	7 21%	13 37%	19 41%	5 14%	0.048
Diabetes mellitus	2 6%	3 9%	2 6%	3 6%	3 8%	0.970
TAA	17 50%	n.d.	35 100%	n.d.	n.d.	n.d.
Bicuspid aortic valve	4 12%	n.d.	5 19% ^§^	n.d.	n.d.	n.d.
hs-TnT [ng/L]	21 (5.4–518)	5.0 (<5.0–11) ^+^	11 (5.1–60)	357 (92–2755)	20 (9.1–71)	<0.001
D-dimers [mg/L]	3.0 (0.6–10.5)	0.2 (<0.2–0.7) ^$^	0.4 (<0.2–3.8) ^$$^	0.4 (<0.2–4.4) ^$$$^	4.0 (1.3–7.1)	<0.001
CRP [mg/L]	5.1 (0.6–136.7)	1.6 (0.4–6.9)	1.1 (0.3–14.5)	3.6 (0.6–36.6)	61.1 (2.0–233.3)	<0.001

For categorical variables, the *p*-value was determined using the χ^2^ test; for continuous variables, using one-way ANOVA. ^§^ data not available for 26% of patients; ^+^ hs-TnT not detectable (<5 ng/L) in 64% of nonCVD; ^$, $$, $$$^ D-dimers not detectable (<0.2 mg/L) in 52% of nonCVD, 17% of TAA and 22% of AMI patients, respectively; n.d. not determined.

**Table 3:** List of OLINK Proseek^®^ Multiplex biomarkers that showed significant difference in the AAD group in comparison to all other analyzed groups.

Short Name	Protein Name	Uniprot Nr.	Proseek Panel	Condition	Median Fold Change	*p*-Value
IL-1ra	Interleukin 1 receptor antagonist protein	P18510	CVD II	nonCVD TAA AMI PE	2.2 3.3 2.0 1.7	1.30 × 10^−5^ 5.27 × 10^−9^ 1.91 × 10^−4^ 0.006
IL-10	Interleukin 10	P22301	INF I	nonCVD TAA AMI PE	7.6 7.1 5.7 6.7	1.58 × 10^−8^ 9.05 × 10^−11^ 2.00 × 10^−6^ 4.53 × 10^−8^
IL-6 *	Interleukin 6	P05231	CVD II INF I ONC II	nonCVD TAA AMI PE nonCVD TAA AMI PE nonCVD TAA AMI PE	15.4 12.1 7.0 2.2 14.0 12.7 7.3 2.2 14.3 12.3 5.7 2.0	1.60 × 10^−10^ 1.11 × 10^−12^ 1.24 × 10^−70^ 0.015 3.65 × 10^−8^ 2.41 × 10^−11^ 5.90 × 10^−7^ 0.031 6.10 × 10^−10^ 2.90 × 10^−12^ 2.95 × 10^−7^ 0.025
IL-1RL2	Interleukin-1 receptor-like 2	Q9HB29	CVD II	nonCVD TAA AMI PE	1.5 1.6 1.5 1.6	0.01 1.45 × 10^−4^ 0.002 0.005
IGFBP1	Insulin-like growth factor-binding protein 1	P08833	CVD III	nonCVD TAA AMI PE	5.2 3.2 3.2 2.2	2.62 × 10^−8^ 0.002 9.70 × 10^−4^ 0.014
PAI1	Plasminogen activator inhibitor 1	P05121	CVD III	nonCVD TAA AMI PE	2.0 2.7 2.8 2.7	7.46 × 10^−4^ 6.68 × 10^−8^ 4.07 × 10^−7^ 2.63 × 10^−8^
TNFB	Lymphotoxin alpha (TNF-beta)	P01374	INF I	nonCVD TAA AMI PE	0.6 0.5 0.7 0.7	1.99 × 10^−7^ 4.88 × 10^−8^ 0.006 0.017

*p*-values determined using one-way ANOVA with Hochberg correction for multiple testing. * IL-6 is included in all three analyzed panels.

**Table 4:** Diagnostic performance for AAD diagnosis of selected biomarkers in the confirmatory cohort.

Biomarker	Area Under Curve (AUC)	95% CI
hs-TnT	0.51	0.41–0.61
D-dimers	0.76	0.68–0.84
IL-10	0.83	0.72–0.94
IL-6	0.75	0.65–0.85
PAI1	0.78	0.67–0.88
IL-1ra	0.71	0.67–0.88
IGFBP1	0.75	0.64–0.85

**Table 5:** Models of discriminant analysis in AAD classification in the confirmatory cohort.

Model		AAD	nonCVD	TAA	AMI	PE	Total
hs-TnT + D-dimers	AAD	9 (26%)	1 (3%)	6 (18%)	4 (12%)	14 (41%)	34 (100%)
	nonCVD	0 (0%)	26 (81%)	6 (19%)	0 (0%)	0 (0%)	32 (100%)
	TAA	1 (3%)	13 (37%)	14 (40%)	2 (6%)	5 (14%)	35 (100%)
	AMI	3 (7%)	0 (0%)	4 (9%)	38 (84%)	0 (0%)	45 (100%)
	PE	11 (31%)	0 (0%)	2 (6%)	0 (0%)	22 (63%)	35 (100%)
		total classification					60%
		AAD sensitivity					26%
		AAD specificity					90%
		**AAD**	**nonCVD**	**TAA**	**AMI**	**PE**	**total**
D-dimers + IL-6 + PAI1	AAD	15 (75%)	0 (0%)	2 (105)	1 (5%)	2 (10%)	20 (100%)
	nonCVD	0 (0%)	20 (72%)	4 (21%)	4 (21%)	0 (0%)	28 (100%)
	TAA	0 (0%)	12 (41%)	6 (21%)	7 (24%)	4 (14%)	29 (100%)
	AMI	1 (6%)	3 (18%)	4 (23%)	8 (47%)	1 (6%)	17 (100%)
	PE	2 (7%)	0 (0%)	1 (4%)	1 (4%)	24 (85%)	28 (100%)
		total classification					60%
		AAD sensitivity					83%
		AAD specificity					95%
		**AAD**	**nonCVD**	**TAA**	**AMI**	**PE**	**total**
hs-TnT + D-dimers + IL-6 + PAI1	AAD	15 (75%)	0 (0%)	2 (10%)	1 (5%)	2 (10%)	20 (100%)
hs-TnT + D-dimers + IL-6 + PAI1	nonCVD	0 (0%)	25 (89%)	3 (11%)	0 (0%)	0 (0%)	28 (100%)
	TAA	0 (0%)	10 (35%)	14 (48%)	2 (7%)	3 (10%)	29 (100%)
	AMI	0 (0%)	0 (0%)	1 (6%)	16 (94%)	0 (0%)	17 (100%)
	PE	3 (11%)	0 (0%)	1 (4%)	0 (0%)	24 (85%)	28 (100%)
		total classification					77%
		AAD sensitivity					83%
		AAD specificity					95%

## Supplementary Materials

The following are available online at https://www.mdpi.com/article/10.3390/diagnostics11040615/s1, Figure S1: Plasma concentrations of IL-1ra (panel A), IL-10 (panel B), IL-6 (panel C), IL-1RL2 (panel D), IGFBP1 (panel E), PAI1 (panel F), TNFB (panel G), D-dimers (panel H), and hs-TnT (panel I) in the exploratory cohort; Figure S2: ROC curves for IL-1ra, IL-6, IL-10, IGFBP1, PAI1, D-dimers and hs-TnT for AAD diagnosis in the confirmatory cohort; Figure S3: Supplemental histochemical and immunohistochemical staining of CD3 and CD19 for both IL-6 and IL-10, CD61 and Ladewig stainig for PAI1 in aortic tissue of patients with AAD; Table S1: Characteristics of primary antibodies used for immunohistochemical staining; Table S2: List of Proseek® Multiplex biomarkers; Table S3: Concentration of IL-1ra, IL-10, IL-6, IGFBP1, and PAI1 in AAD in comparison with nonCVD, TAA, AMI and PE in the confirmatory cohort.

## Abbreviations

AAA	abdominal aortic aneurysm
AAD	acute aortic dissection
AMI	acute myocardial infarction
nonCVD	subjects with non-cardiovascular chest pain
hs-TnT	high-sensitive troponin T
IGFBP1	insulin-like growth factor binding protein 1
IL-1ra	interleukin-1 receptor antagonist
IL-1RL2	interleukin-1 receptor-like 2
IL-6	interleukin 6
IL-1	interleukin 10
PAI1	plasminogen activator inhibitor 1
PE	pulmonary embolism
TAA	thoracic aortic aneurysm
TNF	lymphotoxin-alpha
