# Supplementary material for: Novel Blood Biomarkers for a Diagnostic Workup of Acute Aortic Dissection
Source: Diagnostics (Basel). 2021 Mar 30;11(4):615. doi: 10.3390/diagnostics11040615 (PMC8065878; doi:10.3390/diagnostics11040615)
Supplement: Supplementary file 1 [file diagnostics-11-00615-s001.zip › supplemental figures jgawinecka 110321.docx]

**SUPPLEMENTAL FIGURES**

**Supp. Fig. 1. Plasma concentrations of IL-1ra (panel A), IL-10 (panel B), IL-6 (panel C), IL-1RL2 (panel D), IGFBP1 (panel E), PAI1 (panel F), TNFB (panel G), D-dimers (panel H), and hs-TnT (panel I) in the exploratory cohort**


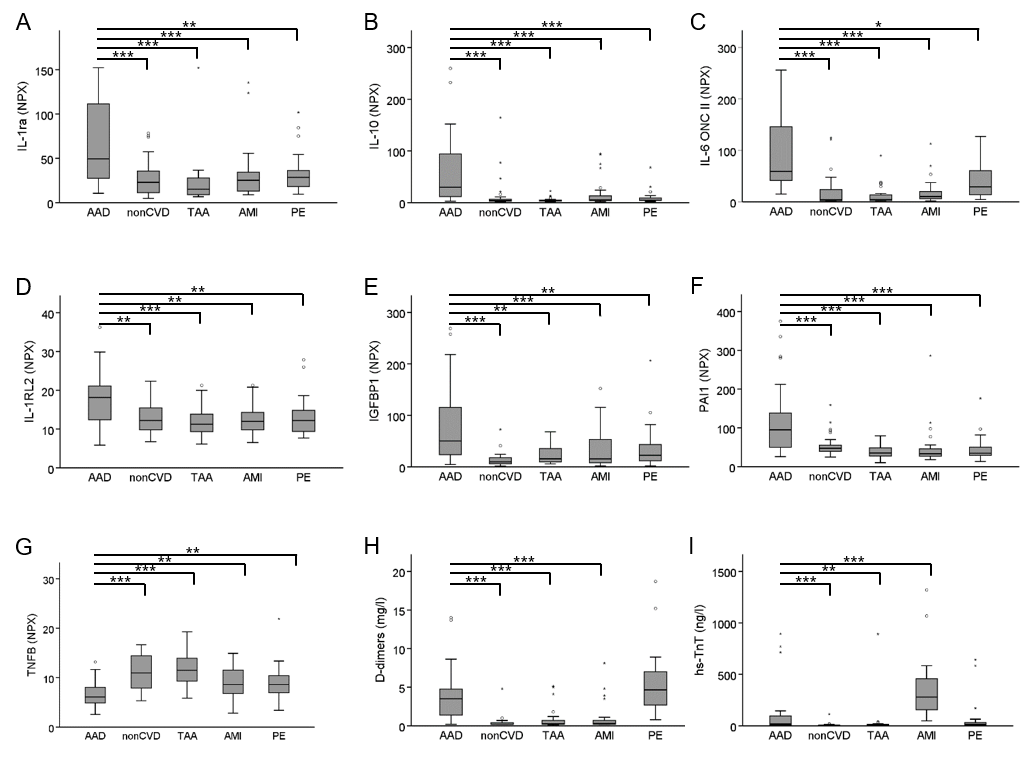


Concentration of IL-1ra, IL-10, IL-6, IL-1RL2, IGFBP1, PAI1 and TNFB are expressed as normalized protein expression (NPX) values. * p-value <0.05; ** p-value < 0.01, *** p-value<0.001

**Supp. Fig 2. ROC curves for IL-1ra, IL-6, IL-10, IGFBP1, PAI1, D-dimers and hs-TnT for AAD diagnosis in the confirmatory cohort**

**
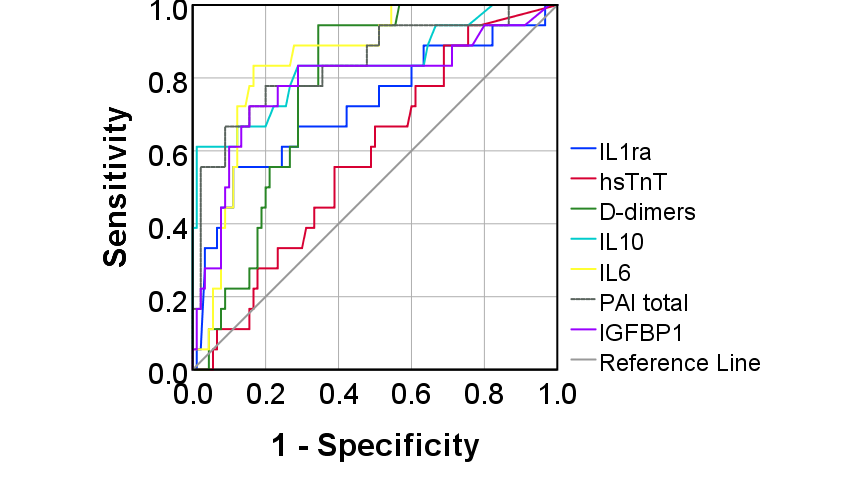
**

**Supp. Fig 3. Supplemental histochemical and immunohistochemical staining of CD3 and CD19 for both IL-6 and IL-10, CD61 and Ladewig stainig for PAI1 in aortic tissue of patients with AAD**

**
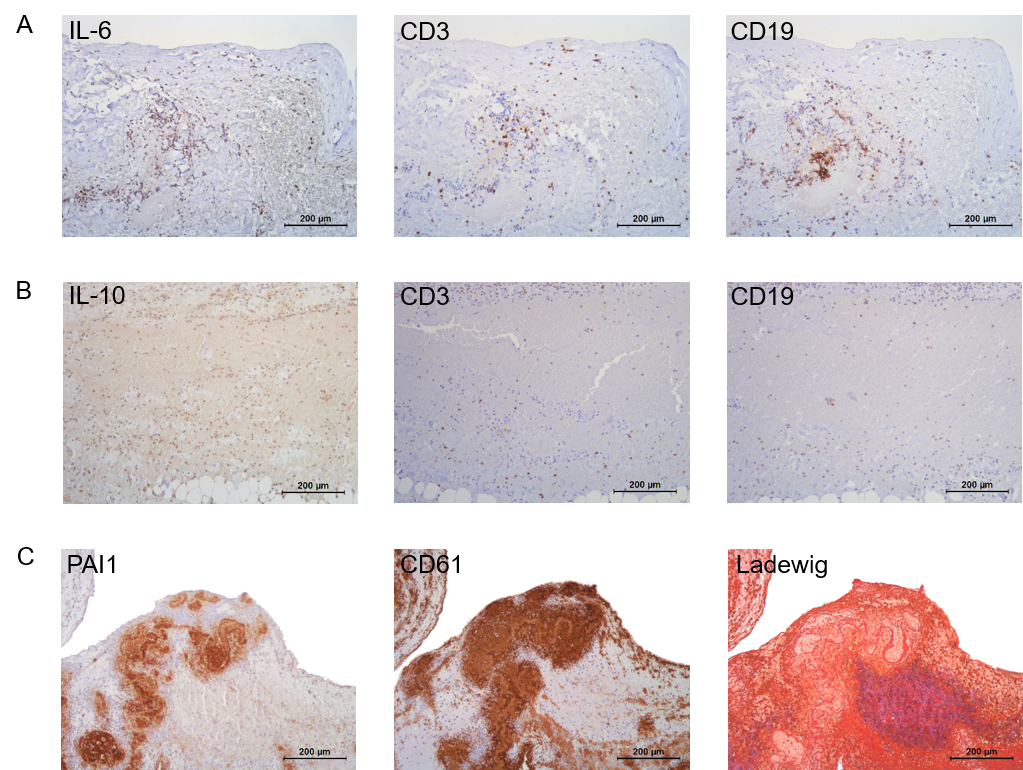
**
