# Supplementary material for: Novel Blood Biomarkers for a Diagnostic Workup of Acute Aortic Dissection
Source: Diagnostics (Basel). 2021 Mar 30;11(4):615. doi: 10.3390/diagnostics11040615 (PMC8065878; doi:10.3390/diagnostics11040615)
Supplement: Supplementary file 1 [file diagnostics-11-00615-s001.zip › supplemental tables jgawinecka 110321.docx]

**SUPPLEMENTAL TABLE**

**Supp. Tab. 1**

**Characteristics of primary antibodies used for immunohistochemical staining**

| **Antibody** | **Company** | **Clone/ Cat. Nr** | **Host species** | **Clonality** | **Reactivity** | **Conjugation** | **Used concentration** |
| --- | --- | --- | --- | --- | --- | --- | --- |
| Anti-IL-1ra | Santa Cruz Biotechnology, Dallas, US | A-11/  sc-376094 | mouse | monoclonal | human | unconjugated | 1:100 |
| Anti-IL-6 | antibodies-online, Aachen, Germany | ABIN6262529 | rabbit | polyclonal | human, mouse, rat | unconjugated | 1:100 |
| Anti-IGFBP1 | antibodies-online, Aachen, Germany | ABIN734948 | rabbit | polyclonal | human, mouse, rat | unconjugated | 1:00 |
| Anti-IL-10 | Abcam, Cambridge, UK | 34843 | rabbit | polyclonal | recombinant fragment | Unconjugated | 1:100 |
| Anti-Serpine1/PAI1 | Covalab, Villeurbanne, France | mab71303 | mouse | monoclonal | human | unconjugated | 1:50 |
| Anti-CD3 | Cell Marque Corporation, Rocklin, US | MRQ-39 | rabbit | monoclonal | human | unconjugated | 1:100 |
| Anti-CD19 | Cell Marque Corporation, Rocklin, US | MRQ-36 | mouse | monoclonal | human | unconjugated | 1:100 |
| Anti-CD163 | Cell Marque Corporation, Rocklin, US | MRQ-26 | mouse | monoclonal | human | unconjugated | 1:50 |
| Anti- Platelet Glycoprotein IIIa / CD61 | Diagnostic BioSystems, Pleasanton, US | Y2/51/Mob164 | mouse | monoclonal | human | unconjugated | 1:100 |
| Anti-CD68 | Dako Agilent, Santa Clara, US | PG-M1 | mouse | monoclonal | human | unconjugated | ready to use |

**The positive staining of following tissues was confirmed in order to demonstrate the suitability of used antibodies: breast cancer for IL-6 (panel A), kidney for IGFB1 (panel B), esophagus for IL-1ra (panel C), colon for IL-10 (panel D), and pancreas for PAI1 (panel E).**


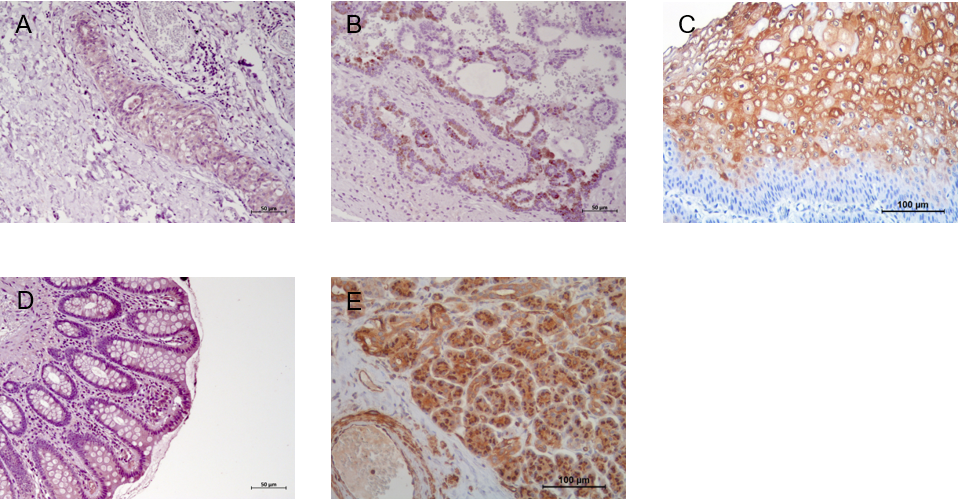


**Supp. Tab. 2**

**List of Proseek® Multiplex biomarkers** *(Excel file)*

**Supp. Tab. 3. Concentration of IL-1ra, IL-10, IL-6, IGFBP1, and PAI1 in AAD in comparison with nonCVD, TAA, AMI and PE in the confirmatory cohort.**

| **Condition** | **IL-1ra** | | | **IL-10** | | | **IL-6** | | | **IGFBP1** | | | **PAI1** | | |
| --- | --- | --- | --- | --- | --- | --- | --- | --- | --- | --- | --- | --- | --- | --- | --- |
|  | **n** | **mg/l** | **p-value** | **n** | **ng/l** | **p-value** | **n** | **ng/l** | **p-value** | **n** | **mg/l** | **p-value** | **n** | **µg/l** | **p-value** |
| **AAD** | 25 | 1.6  (0.6-8.0) | - | 20 | 20.6  (0.7-195) | - | 27 | 41.8  (3.3-107) | - | 27 | 6.9  (0.3-63.4) | - | 27 | 24.3  (7.5-123) | - |
| **nonCVD** | 26 | 1.1  (0.5-2.2) | 0.045 | 32 | 0.6  (0.23-5.3) | 4.50E-05 | 32 | 1.8  (1.5-12.9) | 9.70E-09 | 32 | 1.4  (0.3-9.7) | 0.001 | 29 | 12.2  (8.0-27.9) | 0.024 |
| **TAA** | 28 | 0.9  (0.5-1.6) | 0.005 | 35 | 0.9  (0.1-4.4) | 1.80E-05 | 35 | 5.8  (1.5-28.9) | 7.10E-05 | 35 | 1.2  (0.2-8.7) | 3.60E-04 | 29 | 12.2  (6.7-19.7) | 0.001 |
| **AMI** | 20 | 1.2  (0.4-7.7) | 0.540 | 33 | 1.9  (0.3-17.9) | 0.010 | 35 | 10.6  (3.6-48.4) | 0.181 | 33 | 0.8  (0.2-32.1) | 0.051 | 26 | 9.6  (5.3-38.3) | 0.010 |
| **PE** | 27 | 0.8  (0.2-5.8) | 0.039 | 35 | 0.7  (0.7-5.3) | 0.020 | 34 | 28.8  (4.5-123.2) | 0.988 | 33 | 2.0  (0.3-44.7) | 0.404 | 29 | 7.0  (2.6-16.9) | 2.00E-06 |

Data presented as median with 10^th^-90^th^ percentile in brackets. P-values determined using one-way ANOVA with Games-Howell correction for multiple testing

**Supp. Tab 4. Changes in the mRNA expression of IL-1ra, IL-10, IL-6, PAI1, and IGFBP1 in dissected tissues in comparison to AVR and TAA**

| **Protein**  **name** | **AVR** | | **TAA** | |
| --- | --- | --- | --- | --- |
|  | **log2 change** | **fdr** | **log2 change** | **fdr** |
| **IL-1ra** | 2.37 | 1.67E-07 | 2.34 | 9.56E-05 |
| **IL-10** | 0.49 | 0.020 | 0.046 | 0.028 |
| **IL-6** | 2.72 | 6.69E-05 | 2.85 | 0.002 |
| **PAI1** | 1.81 | 4.45E-07 | 1.24 | 8.59E-07 |
| **IGFBP1** | 0.00 | n.d. | 0.00 | n.d. |

fdr: false discovery rate, n.d. not determined
